# Detecting selection-induced departures from Hardy-Weinberg proportions

**DOI:** 10.1186/1297-9686-41-15

**Published:** 2009-01-21

**Authors:** Joseph Lachance

**Affiliations:** 1 Graduate Program in Genetics, Department of Ecology and Evolution, State University of New York at Stony Brook, Stony Brook, NY 11794-5222, USA

## Abstract

Viability selection influences the genotypic contexts of alleles and leads to quantifiable departures from Hardy-Weinberg proportions. One measure of these departures is Wright's inbreeding coefficient (*F*), where observed heterozygosity is compared with expected heterozygosity. Here, I extend population genetics theory to describe post-selection genotype frequencies in terms of post-selection allele frequencies and fitness dominance. The resulting equations correspond to non-equilibrium populations, allowing the following questions to be addressed: When selection is present, how large a sample size is needed to detect significant departures from Hardy-Weinberg? How do selection-induced departures from Hardy-Weinberg vary with allele frequencies and levels of fitness dominance? For realistic selection coefficients, large sample sizes are required and departures from Hardy-Weinberg proportions are small.

## Introduction

Natural selection modifies the probabilities that alleles are found in either homozygous or heterozygous form. Given that one allele is *A*, what is the probability that the homologous copy of this gene is also *A? *In Hardy-Weinberg populations this is simply equal to *p*, the allele frequency of the *A *allele. When the assumptions of the Hardy-Weinberg principle are violated, such as when viability selection is present, this result cannot be expected to hold. While this has been known for decades, many current studies assume Hardy-Weinberg proportions (*p*^2 ^: 2*pq *: *q*^2^) without explicitly considering the impact of selection. When viability selection results in significant departures from Hardy Weinberg (DHW), the genetic footprint of natural selection can be observed in sequence data [1-3]. Tests of Hardy-Weinberg proportions have been used to detect genotyping errors [4-6]. However, it is an open question whether natural selection confounds such tests. Consequently, one can ask: When does natural selection result in significant departures from Hardy-Weinberg proportions?

Population genetics theory indicates that when fitnesses are non-multiplicative (*w*_*AB*_^2 ^≠ *w*_*AA*_*w*_*BB*_), genotype frequencies differ from Hardy-Weinberg proportions [7]. For example, one expects to only find post-selection copies of a recessive lethal in heterozygotes. While equations describing genotypic frequencies in terms of allele frequencies are deducible for overdominance, mutation-selection balance, and other equilibria, existing theory is lacking when it comes to non-equilibrium populations [8]. There is a need to determine when viability selection leads to significant departures from Hardy-Weinberg proportions [9]. Classical population genetics contains recursion equations that describe post-selection genotype frequencies in terms of pre-selection allele frequencies. However, DHW calculations require allele and genotype frequencies to be from the same time point (*i.e*. post-selection). In this paper population genetics theory is extended, and novel equations are derived for non-equilibrium populations at a single time point. These equations allow the magnitude of viability selection-induced DHW to be quantified and statistical significance to be assessed.

A number of statistical tests of Hardy-Weinberg proportions exist [10-13]. However, these tests do not distinguish between different causes of DHW (such as genetic drift, population subdivision, genotyping error, and natural selection). By coupling population genetics theory to tests from statistical genetics one can determine whether observed departures from Hardy-Weinberg are due to selection. Sample sizes needed to detect selection are found, and they are substantial.

## Methods

### Description of model

A classical population genetics model is used: Hardy-Weinberg plus selection. Consider a single locus with two segregating alleles. Assume that mutation rates are negligible, and generations are discrete and non-overlapping. The population is assumed to be panmictic and large, yielding a deterministic model. Viability selection acts upon zygotes prior to adulthood, with constant genotypic fitnesses denoted by *w*_*AA*_, *w*_*AB*_, and *w*_*BB*_. Genotype frequencies are represented by uppercase letters: *P*_*AA*_, *P*_*AB*_, and *P*_*BB*_. Allele frequencies are represented by lower case letters, with pre-selection allele frequencies in boldface (**p **and **q**) and post-selection allele frequencies in normal typeface (*p *and *q*). After random mating, genotype frequencies are found in Hardy-Weinberg proportions. Genotype frequencies are subsequently weighted by fitness, resulting in the following classic equations from population genetics:

(1a) $$P_{AA}=\frac{p^{2}w_{AA}}{p^{2}w_{AA}+2pqw_{AB}+q^{2}w_{BB}}$$

(1b) $$P_{AB}=\frac{2pqw_{AB}}{p^{2}w_{AA}+2pqw_{AB}+q^{2}w_{BB}}$$

(1c) $$P_{BB}=\frac{q^{2}w_{BB}}{p^{2}w_{AA}+2pqw_{AB}+q^{2}w_{BB}}$$

The above equations can be algebraically manipulated, yielding an equality that contains only post-selection genotype frequencies [14].

(2) $$\frac{{P_{AB}}^{2}}{P_{AA}P_{BB}}=4\frac{{w_{AB}}^{2}}{w_{AA}w_{BB}}$$

Post-selection genotype frequencies are mathematically related to genotype fitnesses [15], and the ratio of genotypic fitnesses in the right hand side of equation (2) can be replaced by a single parameter that represents the extent of fitness dominance (*k*). Note that *k *is always positive.

(3) $$k=\frac{{w_{AB}}^{2}}{w_{AA}w_{BB}}$$

### Post-selection genotype frequencies

Post-selection genotype frequencies differ from Hardy-Weinberg expectations. As per classical population genetics: genotype frequencies sum to one, and allele frequencies are simply weighted genotypic frequencies. These properties, in addition to equation (2), can be combined to obtain post-selection genotype frequencies as a function of post-selection allele frequencies (*p*) and the ratio of genotypic fitnesses (*k*). Factoring with respect to *P*_*AB *_produces a second order polynomial equation:

(4) (1 - *k*)*P*_*AB*_^2 ^+ (2*k*)*P*_*AB *_+ 4*kp*(1 - *p*) = 0

For all possible values of *p *and *k*, the discriminant is positive (*i.e*. solutions of the quadratic equation are real). However, only one root of the quadratic equation produces valid genotype frequencies. The positive root of the quadratic equation results in heterozygote frequencies between zero and one (see equation 6 below). Conversely, the negative root results in *P*_*AB *_< 0 when *k *< 1, and *P*_*AB *_> 1 when *k *> 1. The equations below reduce the description of a post-selection population genetic state to a single allele frequency rather than a collection of genotype frequencies.

(5) $$P_{AA}=p+\frac{k-\sqrt{4p(p-1)k(k-1)+k^{2}}}{2(1-k)}$$

(6) $$P_{AB}=\frac{-k+\sqrt{4p(p-1)k(k-1)+k^{2}}}{1-k}$$

(7) $$P_{BB}=-p+\frac{2-k-\sqrt{4p(p-1)k(k-1)+k^{2}}}{2(1-k)}$$

### Departures from Hardy-Weinberg proportions

Using the above equations, the magnitude of viability selection-induced DHW can be quantified. Multiple measures of DHW exist, with one common measure being Wright's inbreeding coefficient [3,16]. This is equal to one minus the observed heterozygosity over expected heterozygosity.

(8) $$F=1-\frac{P_{AB}}{2pq}$$

Note that genotype and allele frequencies in equation (8) are all post-selection. When *F *is negative there is an excess of heterozygotes, and when *F *is positive there is a deficit of heterozygotes relative to Hardy-Weinberg expectations. Just as inbreeding can lead to DHW, so too can natural selection. Let *F*_*sel *_be a measure of selection-induced DHW. *F*_*sel *_is derived from equations (6) and (8):

(9) $$F_{sel}=1+\frac{k-\sqrt{4p(p-1)k(k-1)+k^{2}}}{2p(1-p)(1-k)}$$

### Statistical measures of DHW

Genotype frequencies in a sample of size *n *need not equal the true genotype frequencies of a population. The observed numbers of each genotype are denoted *n*_*AA*_, *n*_*AB*_, and *n*_*BB *_(where *n*_*AA*_+ *n*_*AB *_+ *n*_*BB *_= *n*). The observed numbers of each genotype in a sample follow a multinomial distribution, and can be used to calculate the magnitude of DHW for a sample ($\hat{F}$):

(10) $$\hat{F}=1-\frac{n\times n_{AB}}{2\times (n_{AA}+\frac{1}{2}n_{AB})\times (n_{BB}+\frac{1}{2}n_{AB})}$$

Given a sample of size *n*, the test statistic *X*^2 ^can be calculated. If sample size is large, *X*^2 ^is conveniently related to *F *[17]. When a null hypothesis of Hardy-Weinberg proportions is true, *X*^2 ^is approximately distributed as a chi-square with one degree of freedom. When a null hypothesis of Hardy-Weinberg proportions is false, *X*^2 ^is approximately distributed as a non-central chi-square [17]. Denoting the non-centrality parameter as *λ*:

(11) *λ *= *nF*^2^

The significance level of a test is equal to α (where α the false positive rate), and the power of test is equal to 1-β (where β is the false negative rate). With one degree of freedom, λ equals 3.84 for an α of 0.05 and a β of 0.5 [18]. Consequently, equation (11) can be rearranged to yield the sample size required to detect selection at a significance level of 0.05 and 50% power.

(12) $$n=3.84{\left(1+\frac{k-\sqrt{4p(p-1)k(k-1)+k^{2}}}{2p(1-p)(1-k)}\right)}^{2}$$

## Results

### Magnitude of selection-induced departures from Hardy-Weinberg proportions

**Figure 1:**
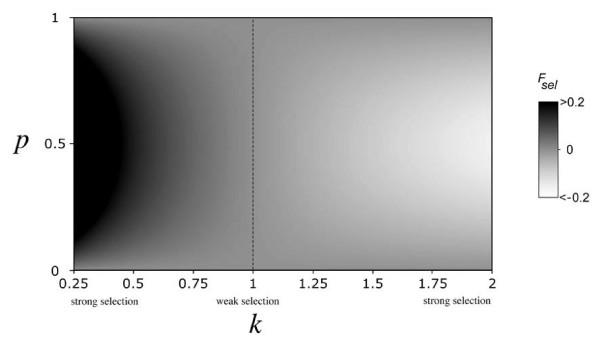
**The magnitude of selection-induced departures from Hardy-Weinberg proportions**. *F*_*sel *_is a function of allele frequency (*p*) and fitness dominance (*k*); negative values of *F*_*sel *_indicate an excess of heterozygotes, while positive values of *F*_*sel *_indicate a deficit of heterozygotes, the dashed line corresponds to Hardy-Weinberg proportions.

The sign and magnitude of selection-induced departures from Hardy-Weinberg are determined by allele frequencies and fitness dominance. Departures from Hardy-Weinberg can be measured by an inbreeding coefficient (*F*_*sel*_). Note that while F-statistics are used, this does not imply that any actual inbreeding is present. Equation (9) describes the magnitude of selection-induced DHW, and *F*_*sel *_is plotted as a function of *k *and *p *in Figure 1. DHW due to viability selection is maximized at intermediate allele frequencies, and minimized when one allele is rare. This is because inbreeding coefficients are relatively insensitive to DHW when minor allele frequencies are close to zero. *k *< 1 results in a deficiency of heterozygotes relative to Hardy-Weinberg expectations, while *k *> 1 results in a surplus of heterozygotes. When *k *takes on intermediate values (*i.e*. selection is weak), *F*_*sel *_is close to zero.

### Large sample sizes are needed to detect selection-induced DHW

To detect selection, sample sizes ranging from thousands to millions are required.

**Figure 2:**
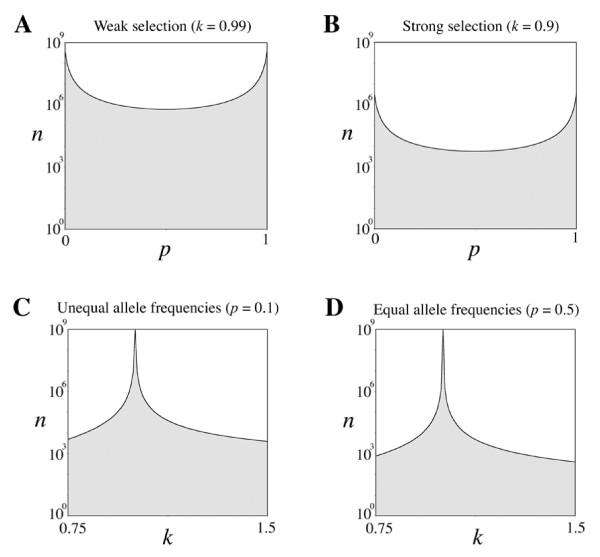
**Sample size as a function of allele frequency and fitness dominance**. Sample sizes (*n*) required to detect selection at a significance level of 0.05 and a power of 0.5 are plotted as a function of allele frequency and fitness dominance; scale on the y-axis is logarithmic; A) Weak selection (*k *= 0.99); B) Strong selection (*k *= 0.9); C) Unequal allele frequencies (*p *= 0.1 and *q *= 0.9); D) Equal allele frequencies (*p *= 0.5 and *q *= 0.5).

In Table 1 sample sizes are listed for multiple types of fitness dominance, allele frequencies, and strengths of selection. Statistical significance is set at 0.05, and power is set at 50%. With the sample sizes indicated, statistically significant selection will still only be detected 50% of the time. Equation (11) indicates that statistical power can be increased above 90% by tripling the sample sizes in Table 1. Note that small sample sizes are more likely to result in observed allele frequencies that differ from the true allele frequencies of a population. When selection coefficients are large (*k *= 0.9), sample sizes on the order of 10^3 ^are required to detect selection. When selection coefficients are small, even larger sample sizes are needed. For example, *k *= 0.99 requires sample sizes on the order of 10^6^. Figure 2 depicts the sample size needed for a range of allele frequencies and selection coefficients. Weak selection and unequal allele frequencies require larger sample sizes, while strong selection and equal allele frequencies require smaller sample sizes. When alleles are found at intermediate frequencies, required sample sizes are largely independent of *p*. The analytic theory used to generate sample sizes was verified by MATLAB simulations. (see Table 2). Here, sample genotype frequencies were drawn via multinomial sampling and tested for significant DHW. This was done 10000 times for each set of parameters, and observed power closely matched expected power.

**Table 1:** Sample size needed to detect selection at 0.05 significance with 0.50 power.

**Fitness dominance**	**Deleterious dominant**	**Deleterious recessive**	**Overdominance**	**Underdominance**
Unequal allele frequencies (*p *= 0.1)				
Weak selection (*s *= 0.01)	4.66 × 10^6^	4.72 × 10^6^	1.21 × 10^6^	1.16 × 10^6^
Medium selection (*s *= 0.05)	1.74 × 10^5^	1.86 × 10^5^	5.30 × 10^4^	4.22 × 10^4^
Strong selection (*s *= 0.1)	3.99 × 10^4^	4.57 × 10^4^	1.48 × 10^4^	9.35 × 10^3^
Equal allele frequencies (*p *= 0.5)				
Weak selection (*s *= 0.01)	6.08 × 10^6^	6.08 × 10^6^	1.55 × 10^5^	1.52 × 10^5^
Medium selection (*s *= 0.05)	2.34 × 10^4^	2.34 × 10^4^	6.46 × 10^3^	5.84 × 10^3^
Strong selection (*s *= 0.1)	5.54 × 10^3^	5.54 × 10^3^	1.69 × 10^3^	1.39 × 10^3^

α = 0.05 and β = 0.5; sample sizes are computed using equation (12); fitness dominance parameters are as follows: deleterious dominant *k *= 1 - *s*, deleterious recessive allele *k *= 1/(1 - *s*), overdominance *k *= (1 + *s*)^2^, underdominance *k *= (1 - *s*)

**Table 2:** Verification of analytic theory via MATLAB simulation.

**Allele frequency (*p*)**	**Fitness dominance (*k*)**	**Sample size (*n*)**	**Significance (α)**	**Expected power (1-β)**	**Observed power (simulated)**
0.5	0.9	5537	0.05	0.5	0.4942
0.5	0.9	15148	0.05	0.9	0.9003
0.1	0.9	39944	0.05	0.5	0.4947
0.1	0.9	109222	0.05	0.9	0.8971

Sample sizes were obtained from equations (11) and (12); for each parameter set, true post-selection genotype frequencies were obtained from equations (5), (6), and (7); sample genotype counts were then generated via multinomial sampling, and chi-square tests were performed; MATLAB simulations were run 10000 times for each parameter set, and the proportion of tests that resulted in detectable DHW were recorded.

## Discussion

### Magnitude of selection-induced departures from Hardy-Weinberg proportions

For moderate levels of fitness dominance (*i.e. k *close to one), the magnitude of *F*_*sel *_is small. Consequently, Hardy-Weinberg proportions reasonably approximate post-selection genotype frequencies. As a point of comparison, a population containing an uncommon (*p *= 0.1) completely dominant allele that reduces viability by 1% has the same magnitude of DHW as a population where every mating involves 4^th ^cousins (*F *≈ 0.0009). In the context of forensic genetics, the National Research Council set notable levels of DHW at *F *> 0.01 for cosmopolitan populations [19]. Given an actual *F *of this magnitude, a sample size of 38400 would be required to reject a null hypothesis of *F *= 0 (α = 0.05, β = 0.5).

An interesting property of Hardy-Weinberg Equilibrium is that one can infer complete single-locus genotypic states from partial data (*i.e*. one can infer *P*_*AB*_, *P*_*BB*_, *p*, and *q *from *P*_*AA*_). This also holds for post-selection frequencies in a one-locus, two-allele system. An exception involves heterozygote frequency data (which maps to a pair of possible allele frequencies). Given genotypic fitnesses and single genotype frequency, *p *can be found via equation (5), (6), or (7). Subsequently, *p *and *k *can be used to obtain the post-selection frequencies of other genotypes. In practice, however, one is much more likely to have complete genotype frequency data than complete knowledge of genotypic fitnesses.

### Large sample sizes are required to detect selection-induced DHW

Statistically significant DHW requires large departures from neutrality and is maximized at intermediate allele frequencies. For example, a sample size of 1000 is too small to reliably detect significant DHW for a recessive gene that confers a 20% fitness advantage (*i.e*. power is less than 0.5 for *p *= 0.5, *k *= 0.83, α = 0.05, and *n *= 1000). As shown in Figure 2, sample sizes become prohibitively large when *k *is close to one. It is known that non-central chi-square tests can over-estimate statistical power when alternative hypotheses differ greatly in their expectations [20]. However, selection-induced departures from Hardy-Weinberg proportions are of small magnitude. As verified by MATLAB simulations, equations (11) and (12) accurately determine the sample size needed to detect selection-induced DHW.

### Implications

If only two alleles are segregating, heterozygosity tests of neutrality require large sample sizes [21,22]. Many alleles are nearly neutral [23], with values of *k *close to one. However, the scope of undetectable selection extends over a much wider range of parameter space than the range of nearly neutral genes. DHW is a poor indicator of natural selection in the wild. This qualitative conclusion is unlikely to be changed when the assumptions of this paper's model are relaxed. Mutation, assortative mating, and finite population size are all likely to further obscure the signature of selection on genotype frequencies. Also note that genes under directional selection are less likely to be observed at intermediate allele frequencies (*i.e*. frequencies favourable to the detection of significant DHW).

A lack of significant DHW does not imply neutrality. There are large regions of parameter space where viability selection can lead notable changes in allele frequencies over time without producing significant DHW in any single generation. Multiple mechanisms can result in a failure to detect selection even when it is present (*i.e*. there is a type II error). For example, population structure can modify genotype frequencies, masking the effects of selection. Evolutionary geneticists are more likely to detect the footprint of natural selection via use of multilocus linkage disequilibrium data and Poisson random field models [24,25]. Positive selection results in linkage disequilibrium adjacent to the selected locus, the extent of which can be used to estimate the age of alleles. While genotype frequencies at a single locus can be used to detect selection in the most recent generation, linkage disequilibrium data bears the footprint of past selection. Alternatively, natural selection can be measured over multiple generations in the wild [26] or via experimental evolution studies. If genotype frequencies are obtained from wild populations, care must be taken to ensure that genotyped individuals share the same age.

## Competing interests

The author declares that they have no competing interests.

## Authors' contributions

JL designed the study, performed all statistical analyses and wrote the paper.
